# Death by Chloroform Prevented by Electricity

**Published:** 1871-01

**Authors:** 


Death by Chloroform Prevented by Electricity.—On the 22d of November, 1866, Dr. Danzel administered chloroform for the removal of a cancer. After ths operation the patient ceased to breathe, and opening the windows, artificial respiration, and all other agencies proved of no avail, when recourse was had to electricity. One pole of the battery was applied to the neck, the other to the epigastrium. There was soon a movement of the muscles, and by degrees the respiration was restored. There is no doubt that death would have ensued without the application of electricity, and as this remedy has been applied with success in several other cases, it is worthy of note on the part of physicians generally.—*Journal of Applied Chemistry.*

